# Nitrogen and Stem Development: A Puzzle Still to Be Solved

**DOI:** 10.3389/fpls.2021.630587

**Published:** 2021-02-15

**Authors:** Lucas Anjos Souza, Rafael Tavares

**Affiliations:** ^1^Innovation Centre in Bioenergy and Grains, Goiano Federal Institute of Education, Science and Technology, Goiás, Brazil; ^2^Department of Cell and Development Biology, John Innes Centre, Norwich Research Park, Norwich, United Kingdom

**Keywords:** stem development, nitrogen use efficiency, internode elongation, high crop yield, nitrogen fertilizer

## Abstract

High crop yields are generally associated with high nitrogen (N) fertilizer rates. A growing tendency that is urgently demanding the adoption of precision technologies that manage N more efficiently, combined with the advances of crop genetics to meet the needs of sustainable farm systems. Among the plant traits, stem architecture has been of paramount importance to enhance harvest index in the cereal crops. Nonetheless, the reduced stature also brought undesirable effect, such as poor N-uptake, which has led to the overuse of N fertilizer. Therefore, a better understanding of how N signals modulate the initial and late stages of stem development might uncover novel semi-dwarf alleles without pleiotropic effects. Our attempt here is to review the most recent advances on this topic.

## Introduction

To secure steadily growing global demand for food, agronomic practices have increasingly spurred more nitrogen (N) fertilizer inputs to agricultural lands, leading not only to economic competitiveness between smallholder farmers, but also causing detrimental and pervasive impacts on the environment and climate (Cui et al., 2018; Kanter et al., 2019). Yet according to FAO (2019), N fertilizer consumption may continue its uptrend on global demand in the foreseeable future, rising by 2.6% to reach 111.5 teragrams (Tg) N by 2020/2022. In a world of climate volatility and over-farming, global food security is reliant on crop yield forecasting, which entails various elements of uncertainty and necessity that might lead to the over application of N. At the farm level, for instance, lack of information about the bountiful supply of N available in the soil (Ladha et al., 2016; Yan et al., 2020) as well as precision agriculture (Omara et al., 2019) have led to uncertainties about N application rates by farmers (Lobell, 2007). Optimization of N dosage through site-specific best management practices (BMPs) has been proposed as the sustainable agriculture flagship to prevent run-off, which accounts for 67% of applied N fertilizer for cereal production worldwide (Raun and Johnson, 1999). On the other hand, the necessity for high N input has been a determinant factor, whereas the main cereal crops present a low nitrogen use efficiency (NUE) that demands considerable amounts of N for food production needs (Hawkesford and Griffiths, 2019). In the past, particularly in rice and wheat, breeders altered the growth response to N through the introduction of semi-dwarf genes to shorten the plant stature, the so-called Green Revolution (GR) varieties (Peng et al., 1999; Spielmeyer et al., 2002). As a result, they were able to reduce the lodging risk (i.e., bend or break the stem base), and to maximize yield potential in these modern varieties (Ortiz-Monasterio et al., 1997; Gooding et al., 2012); but as cited above, it has caused an unprecedented “domino effect” of N inputs, owing to the negative pleiotropic effects such as poorer N uptake (Li et al., 2018; Hawkesford and Griffiths, 2019; Wang et al., 2020; Wu et al., 2020).

Therefore, in parallel with BMPs and precision agriculture, the attenuation or elimination of the necessity of high N input must be targeted in the modern cereal crops. Although these high-yielding semi-dwarf varieties present an improved N utilization efficiency (i.e., grain yield per unit of N uptake) due to the direct response to fertilizer inputs without the effect of lodging, on the other hand, their N uptake efficiency (i.e., the capacity of the roots to acquire N from the soil) is negatively compromised by the dual-faceted impacts of gibberellin (GA) on plant height and N uptake (Li et al., 2018; Wu et al., 2020). Despite recent contributions on N uptake should be pointed out (Feng et al., 2020; Rahikainen and Kangasjärvi, 2020), this mini-review attempts to summarize the current knowledge of how N regulates stem development, in order to encourage progress toward better semi-dwarfing alleles without undesirable effects in the future.

## Nitrate Is a Driving Force of the Initial Stage of Stem Development

**Figure 1:**
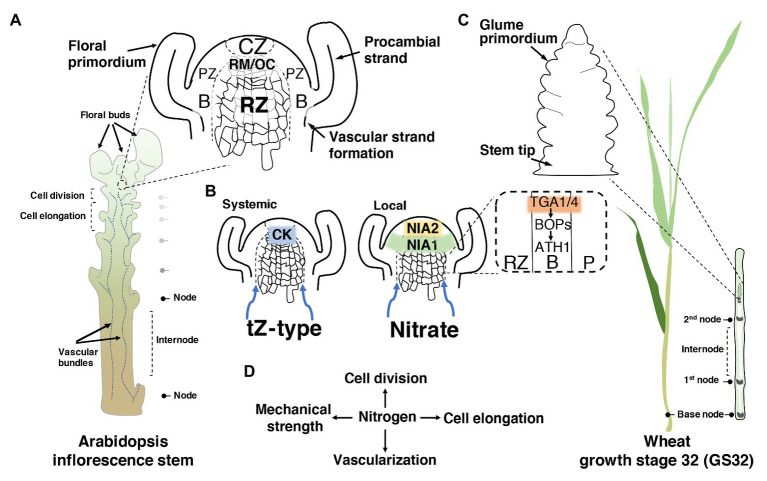
How nitrogen (N) may modulate stem development. **(A)** Schematic illustration of *Arabidopsis* inflorescence stem showing the longitudinal section of the shoot apical meristem (SAM) and the meristematic zones. In detail, a representation of the oriented cell division in the rib zone (RZ). CZ, central zone; RM/OC, rib meristem/organizing centre; PZ, peripheral zone; B, organ boundary. **(B)** The dual nitrogen sensing within the shoot apex through a systemic root-to-shoot transport of the active cytokinin (CK) trans-zeatin (tZ), and the local sensing of nitrate through the nitrate reductase enzymes. In the dashed square, the two regulatory factors of the primary nitrate responses (TGA1/4) are highlighted, evidencing their involvement in the activation of the organ boundary genes. Blue arrows show the direction of root-to-shoot transportation. **(C)** Schematic illustration of wheat showing the growth stage 32 (GS32) based on Zadoks et al. (1974). In wheat, most N is taken up during the stem elongation phase (GS30–GS37) until the flowering stage. On the right, the longitudinal section of the nodes and internodes, and the floret initiation at this stage. **(D)** A simplified scheme of the central role of N in the regulation of the four different aspects of stem development.

All aerial organs are initiated at the apical dome, also known as shoot apical meristem (SAM). This region comprises dynamic and spatially functional zones that provide robustness and plasticity during the entire shoot ontogeny. Broadly speaking, at the tip of the SAM, the central zone (CZ) moves continuous daughter cells into the rib meristem (RM), where the stem’s central core (pith) originates, and into the surrounding peripheral zone (PZ), which contributes to the stem epidermis and cortex (Gaillochet et al., 2015). Besides, the peripheral and central rib regions tandemly locate together, forming the rib zone (RZ), where proliferation and expansion give rise to the axial elongation in seed plants (McKim, 2019; Figure 1A). Thus, a sophisticated interconnection network between the zones through metabolites, non-cell-autonomous proteins, and phytohormones controls the size of the meristem and the rate of shoot organogenesis, ensuring a robust, plastic developmental spectrum (Tian et al., 2019).

This raises the question of whether there is a precise N-led signaling pathway, or it is the spreading of N to the zones of the SAM that modulates RZ activity and stem elongation. Currently, novel pieces of evidence in *Arabidopsis thaliana* (hereafter called *Arabidopsis*) suggest the coexistence of dual N sensing within the SAM: a systemic and local signal. The systemic signal relies on the activation of trans-zeatin (tZ)-type cytokinin (CK) in roots, in response to N supply, and its translocation to the SAM *via* the xylem (Landrein et al., 2018; Poitout et al., 2018). Impairing CK allocation to the SAM through the *cyp735a1 cyp735a2* double mutant, in which tZ-type CKs are severely reduced, exhibited a shortened inflorescence stem similar to that of *abcg14*, an important gene for CK transport (Kiba et al., 2013; Poitout et al., 2018). Notably, tZ content may also positively influence glutamate/glutamine levels, which are known to promote stem elongation (Poitout et al., 2018). In contrast, the local signal comprises the action of nitrate itself entering into the SAM, where it is assimilated in the RZ and the organ boundary domain (B) through the nitrate assimilatory enzymes nitrate reductases (NIA1 and NIA2; Olas et al., 2019; Figure 1B).

The notion that these enzymes act as an N-sensitive checkpoint in the SAM may be corroborated by the fact that their expression and activity are highly regulated to fine-tune the sensing and integration of carbon (C)/N ratio (Klein et al., 2000; Park et al., 2011; Kim et al., 2018). The balance that is also determined by the tricarboxylic acid (TCA) cycle intermediate 2-oxoglutarate (2-OG), which is the major carbon skeleton in N-assimilatory reactions for the synthesis of glutamate (Zheng, 2009; Huarancca Reyes et al., 2018). Interestingly, boundary domains exert a critical function in preserving stem elongation, whereas an ectopic expression of boundary genes [BLADE-ON-PETIOLE1 (BOP1/2)-*Arabidopsis thaliana* HOMEOBOX1 (ATH1)-KNOTTED1-LIKE FROM A.THALIANA6 (KNAT6)] in the RZ causes growth defects (Khan et al., 2012a,b; Hepworth and Pautot, 2015). Recent work has shown that TGACG-motif binding-1 and -4 (TGA1/4), two regulatory factors of the primary nitrate responses (Alvarez et al., 2014), interact and recruit BOP1/2 coactivators to the promoter of ATH1 homeobox in *Arabidopsis* (Wang et al., 2019; Figure 1B). ATH1 is known to repress RZ proliferation, whereas *ath1-3* mutants displayed longer internodes than the wild-type control (Gómez-Mena and Sablowski, 2008). Scrutinizing the potential of this integration of TGA1/4 in N response and stem growth might open up new avenues for NUE.

Similar to other organs, stem development is regulated by the activity of two combined actions: cell division and expansion. In the most apical region of the RZ in both dicots and monocots [plus the intercalary meristem (IM) in grasses, the details are below] lies the active cell division which is regulated by GA (Sachs et al., 1959; Sachs, 1965; Serrano-Mislata et al., 2017). The notorious close interrelation between GA stimuli and N homeostasis at different regulatory levels in plants (David et al., 2016; Gras et al., 2018; Wang et al., 2020) creates a compelling logic to consider other semi-dwarfing alleles influencing stem elongation due to the negative pleiotropic effects. Strikingly, almost 67% of GA-regulated genes in *Arabidopsis* require brassinosteroids (BRs; Bai et al., 2012). This high dependence reflects the interaction network of BR and GA at multiple levels in model plants that could be further explored for NUE. In rice, for instance, the brassinosteroid deficient mutant (*osdwarf4-1*) presented a slightly dwarfed stature and more erect leaves, which enhanced biomass production and grain yield, without extra fertilizer (Sakamoto et al., 2006).

Besides, recent studies have demonstrated the involvement of the microRNA miR396/growth regulating-factors (GRFs)/GRF-interacting factors (GIFs) regulatory module in the interaction network of BR and GA signaling (Tang et al., 2018; Zhang et al., 2020). The overexpression of miR396 represses organ growth in *Arabidopsis* by repressing the activity of the targeted GRF and GIF genes (Rodriguez et al., 2010). Interestingly, the miR396 acts downstream of DELLA, the negative regulator of GA responses, and upstream of GA-induced cell-cycle genes for the control of stem elongation in rice (Lu et al., 2020). Conversely, more recently, the miR396e and miR396f (*miR396ef*) rice mutants showed an increased grain yield under nitrogen-deficient conditions (Zhang et al., 2020). Future studies addressing the cross-talk between N and BR signaling and the miR396-GRFs module in the RZ may disclose a new perspective on N-driven stem elongation.

At the early stages of stem development, the establishment of a vascular pattern is an important aspect. New vascular strands are initiated by the canalization of auxin flow from new primordia toward a pre-existing vascular network (Scarpella, 2017). As auxin signaling is inhibited in the RM region in dicotyledons, these new vascular networks are initiated at the boundary between the peripheral and the central regions of the RZ (Banasiak et al., 2019). Reflecting on the importance of auxin in controlling the formation of veins and their connections, recent work revealed the uniform expression of TRANSPORT INHIBITOR RESPONSE1 (TIR1)/AUXIN-SIGNALING F-BOX (AFB) proteins in the SAM (Prigge et al., 2020). AFB3 is directly regulated by nitrate (Vidal et al., 2010) and potentially regulates the direction of auxin transport during stem vascularization (Wulf et al., 2019). Besides, two master regulators of primary nitrate response, NIN-LIKE PROTEIN6/7 (NPL6 and NPL7) are expressed in the SAM and adjacent PZ (Olas et al., 2019). Notably, recent findings connect NLP7 with the Ca^2+^-sensor protein kinases (CPKs) to orchestrate nutrient-growth regulatory networks (Liu et al., 2017). Although CPK28 is not part of the subgroup III, of which the genes are nitrate-responsive (e.g., CPK10, CPK30 and CPK32), it controls stem elongation and vascular development in *Arabidopsis* (Matschi et al., 2013). Further studies will be required to scrutinize in more detail their roles in stem development.

Moreover, once a stem starts growing (i.e., N-demanding tissue), vascularisation plays an essential role in the source-to-sink N remobilization (Fernie et al., 2020). As such, nitrate transporters on major and minor veins facilitate N allocation to fast-growing sinks, optimizing plant growth in N-sufficient and N-deficient conditions (Tegeder and Masclaux-Daubresse, 2018; Chen et al., 2020). One example is the nitrate transporter1/peptide transporter family (*osnpf2.2*) rice mutants, which showed growth retardation and abnormal vasculature (Li et al., 2015). Apart from inorganic N, organic N might also be critical for stem development. For instance, polyamines (putrescine, spermidine and spermine) are aliphatic amines that act as growth regulators in plant growth and development (Chen et al., 2019). It is worthwhile to investigate the increase of polyamine content in nitrate and ammonium-grown plants (Garnica et al., 2009; Paschalidis et al., 2019), whereas the ACAULIS5/THICKVEIN (ACL5/TKV) protein, a thermospermine synthase, is also involved in stem elongation and vascularization in plants (Hanzawa, 2000; Clay and Nelson, 2005; Vera-Sirera et al., 2015).

## N Signaling in the Later Stages of Stem Development: Internode Elongation and Lignification

In dicot plants with a rosette habit such as *Arabidopsis*, radish and cabbage, among other species, the compressed vegetative internodes shift to an acropetal expansion after the reproductive transition. In contrast, in monocots, particularly grasses, vegetative internodes are promoted by intercalary meristems (IMs), located at the base of each internode (Figure 1C). After an increased mitotic activity within the IMs, the cells are displaced upward, entering various zones of expansion and lengthening each succeeding internode until the heading stage, which later gives rise to the grain-laden inflorescence (McKim, 2020).

Thus, both RZ and internodal regions exhibit various sorts of cells differing in their states of proliferation, growth, and differentiation. Regarding cell growth, for instance, a high level of endopolyploidy (i.e., modified cell cycle without cytokinesis) occurs in pith cells following organ maturation in *Arabidopsis* and maize (Jacqmard et al., 1999, 2003; Li et al., 2019). This ties in with a recent study showing that nitrate signaling regulates shoot growth by controlling endoreduplication through the upregulation of a key cell cycle regulatory gene *LGO*, a known cyclin-dependent kinase (CDK) inhibitor (Moreno et al., 2020). Given that nitrate regulates LGO-mediated endoreduplication and cell expansion in *Arabidopsis*, it is reasonable to speculate whether such modulation is also present within the RZ and internodal regions of cereal and bioenergy crops, which may also explain the N-responsive stem elongation of such crops (Euring et al., 2014; Zeng et al., 2020).

In addition, cell proliferation and expansion strictly depend on the mechanical properties of primary cell walls (CW). Differences in the expression of CW-related genes and CW composition have been observed during stem elongation (Hall et al., 2013; Hall and Ellis, 2013). A detailed study of CW composition and the dynamic and mechanical properties of the *Arabidopsis* inflorescence stem suggested that changes in the pectin structure, dynamism and mobility lead to weak pectin-cellulose interaction, being likely the main factors leading to the wall extensibility in fast-growing regions (Phyo et al., 2017). Indeed, CW analysis of the upper region of the stem (high growth intensity) presented higher pectin and lower amounts of xyloglucan (XyG) and (lower) cellulose contents (Phyo et al., 2017). Of interest, in type I-CW, in which XyG is the most abundant hemicellulose, a very recent study of *Arabidopsis* showed that the cell wall-related gene xyloglucan endotransglucosylases-9 (XTH9), which is highly expressed in the shoot apices and might contribute to cell elongation in the stem (Hyodo et al., 2003), is regulated by the nitrate signaling pathway (Xu and Cai, 2019).

Moreover, although the specific mechanisms are still to be understood, novel evidence suggests that cellulose content is modulated in response to N status in *Arabidopsis* and rice (Landi and Esposito, 2017; Zhang et al., 2017). Yet, in grass-specific type-II CW, different inorganic N forms, such as nitrate and ammonium, may modify the chemical structure of pectins and hemicelluloses (Podgórska et al., 2017). The CW properties are thereby dynamically regulated to allow sufficient nutrients to reach demanding organs, as well as to allow cell expansion prior to growth cessation, a tightly regulated process that is accompanied by N status (Głazowska et al., 2019).

Stem maturation is followed by secondary cell wall production and lignification (Barros et al., 2015), which confer stem properties such as length, flexibility and strength, and are tightly regulated to prevent bending and breaking which lead to crop lodging. Interestingly, recent work showed that high N availability substantially reduces the H, G and S monolignol precursors (*p*-coumaryl alcohol, coniferyl alcohol, and sinapyl alcohol, respectively) of lignin, and hence, the total lignin content in the shoot of maize seedlings (Sun et al., 2018). The authors elegantly demonstrated that the miR528, a monocot-specific miRNA expressed in vascular tissues, is upregulated by N supply, leading to the repression of *ZmLACCASE3* (*ZmLAC3*) and *ZmLACCASE5* (*ZmLAC5*), oxidative enzymes involved in lignin polymerization (Schuetz et al., 2014), that will ultimately make such plants more prone to lodging under high N supply (Sun et al., 2018). Likewise, a high N supply increases the lodging index in two varieties of japonica rice, owing to the significant reduction of cellulose and lignin contents (Zhang et al., 2017). Yet, CW profiles of *Brachypodium* supplied with different types of N source (ammonium and nitrate) showed that nitrate-fed plants were prone to less lignification rates than those from ammonium-fed plants, suggesting that the CW architecture is modulated according to the uptake and assimilation of different N form through the cross-talk between N metabolism and CW synthesis (Głazowska et al., 2019).

These recent results demonstrated that cell expansion (plastic growth) and CW lignification are strictly influenced by N availability. A key mechanism that might be coordinating these adaptive changes is the cell wall integrity (CWI) maintenance mechanism that is conserved in both monocot and dicot plants (Bacete and Hamann, 2020). Intriguingly, recent results from genetic analyses suggest that NIA1 and NIA2 act downstream of THESEUS1 (THE1), a surface CW sensor, in initiating CW damage responses (Gigli-Bisceglia et al., 2018). THE1 is expressed in elongating cells and in vascular tissues in *Arabidopsis*. Among its target genes, various CW-related proteins involved in loosening and stiffening are regulated, such as extensins, peroxidase 59 and expansin 1 (Hématy et al., 2007). Thus, the CWI mechanism might be a regulator of plant growth according to N status from the environment. Future investigations may unveil this intricate action of N into CWI signaling, which might be a potential target for heightening NUE in crops.

## Conclusion and Perspectives

Although a shorter stature and stem sturdiness have revolutionized world cereal production in the last 50 years, the adoption of the original semi-dwarfing alleles has also brought the necessity for an increasing amount of N fertilizer due to the negative pleiotropic effects. Although several studies have been carried out to understand the genetic basis of N assimilation, curiously, very little attention has been paid so far to how environmental N signals modulate RZ activity and stem development, mainly in monocot plants (Figure 1D). With the advent of recent technical advances in quantitative imaging (Serrano-Mislata et al., 2017), bio-imaging (de Reuille et al., 2015), and biophysical techniques (Phyo et al., 2017; Shah et al., 2017), along with developmental genetics, a clear picture of molecular, cellular and mechanical mechanisms of stem growth is increasingly emerging. Understanding these developmental mechanisms will allow more genetic tools to alter stem architecture and eliminate the root cause of high N need in modern semi-dwarfing varieties in order to increase productivity and decrease environmental pollution.

## Author Contributions

LAS and RT conceived, discussed, organized and contributed equally for writing and reviewing all this manuscript. All authors contributed to the article and approved the submitted version.

### Conflict of Interest

The authors declare that the research was conducted in the absence of any commercial or financial relationships that could be construed as a potential conflict of interest.
